# Treatment of Extramedullary Myeloid Sarcoma With Radiotherapy

**DOI:** 10.7759/cureus.15676

**Published:** 2021-06-15

**Authors:** Suzanne R Graham

**Affiliations:** 1 Radiation Oncology, Genesis Care, Bundaberg, AUS

**Keywords:** acute myeloid sarcoma, orbital swelling, acute myeloid leukemia (aml), radiotherapy (rt), palliative radiation therapy

## Abstract

Myeloid sarcoma is a rare pathology with important clinical implications. In this paper, we report the case of a 95-year-old gentleman with an orbital mass, which was later confirmed to be a myeloid sarcoma. We also discuss the role of radiotherapy in regard to this diagnosis in patients.

## Introduction

Myeloid sarcoma (also known as chloroma, granulocytic sarcoma, or myeloblastoma) is a rare extramedullary manifestation of acute myeloid leukaemia (AML) with an estimated incidence throughout the literature of under 1% [1]. This rare neoplastic condition, which consists of immature myeloid cells, most often occurs in areas such as bone and skin; however, almost any area of the body can be affected. It can sometimes be the first manifestation of AML in a patient, precede a diagnosis of AML, or even be the initial finding in a patient who has relapsed from previously treated AML [2,3].

## Case presentation

Our patient was a 95-year-old gentleman from a nursing home background who presented with a large, fixed, non-tender mass around his left orbit, which progressed quickly over the two-week period prior to presentation. His vision was intact, but the pressure of the mass was causing discomfort to the patient. He had obvious proptosis of the left eye and associated diplopia as the mass progressed in size.

The patient had a history of several non-melanomatous skin cancers in the past, which had been managed by his general physician, but no history of any haematological issues or other relevant medical history.

**Figure 1:**
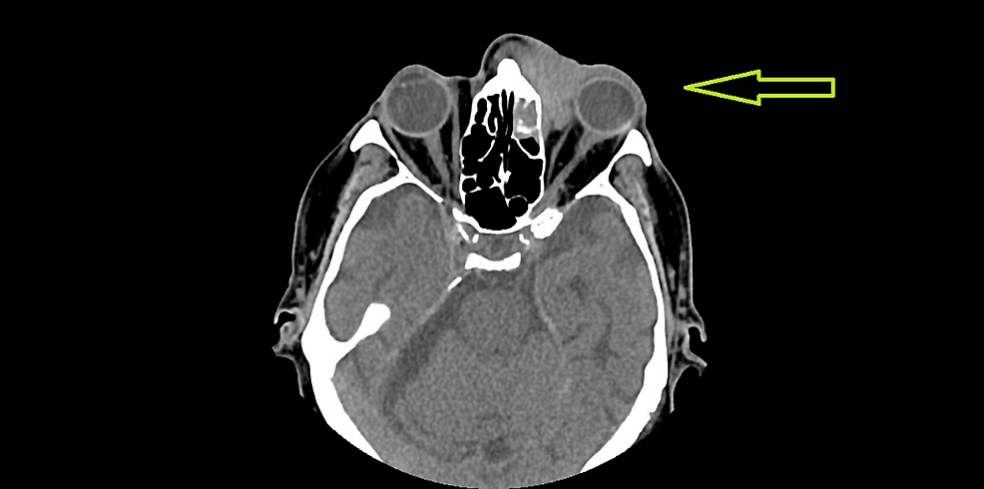
CT scan showing orbital mass affecting medial rectus muscle.

When he first presented, it was felt to be an orbital cellulitis and treated with IV antibiotics, which did not result in any clinical improvement. A CT was then performed, which showed a mass that was invading his left orbit. It was noted that the mass extended into the left orbit from the medial side, displacing the eye and medial rectus muscle laterally (Figure 1). No optic nerve involvement was seen on the CT and the mass did not encroach on the ophthalmic vessels. No destruction of bone was seen. 

The decision was made to admit the patient to hospital for further investigations. A punch biopsy was performed, which showed extensive infiltration of the dermis, subcutis, and skeletal muscle by a neoplastic infiltrate of epithelioid cells, with decohesion. The appearance of the biopsy and the immunohistochemical profile were in keeping with myeloid sarcoma.

The patient’s case was discussed with both an ophthalmic surgeon as well as a haematology consultant. The conclusion was that this was in keeping with a manifestation or prelude to AML. It was agreed, in conjunction with the patient, that the best approach to treatment in this 95-year-old gentleman would be palliative radiation to the affected site. It was also decided not to proceed with further invasive investigations such as a bone marrow biopsy. 

Further CT scanning for staging showed no evidence of primary malignancy, metastases, or lymphoma. Blood tests showed no abnormalities except a mild hyponatraemia. Blood film was unremarkable.

It was decided to treat him with a course of 30 Gy in 10 fractions using volumetric modulated arc therapy (VMAT) with bolus.

**Figure 2:**
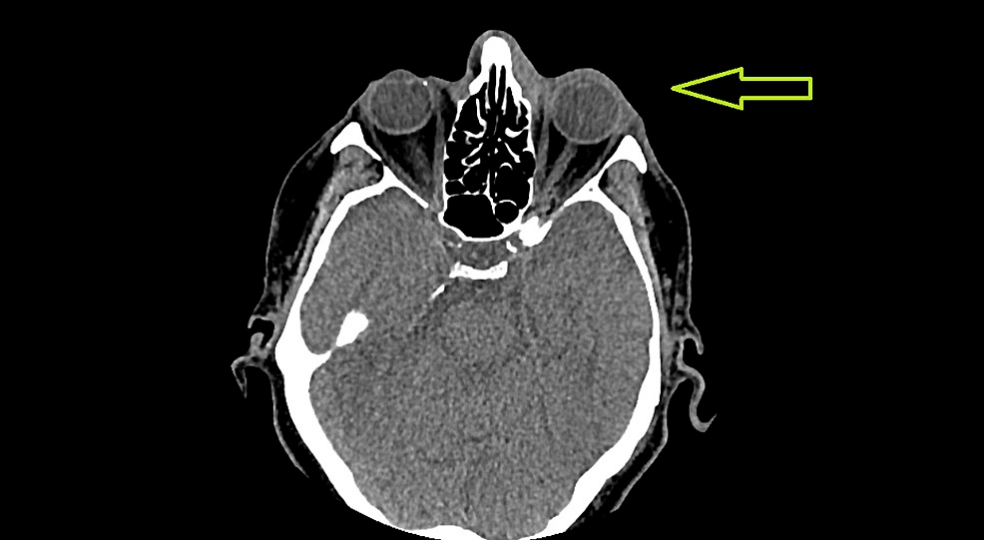
CT scan after radiotherapy to the affected area, showing marked improvement.

Throughout the treatment, he showed a marked clinical improvement with a visible decrease in degree of proptosis and size of mass (Figure 2). The patient also reported less pressure symptoms than before as well as the resolution of diplopia. On his seventh treatment, it was decided to end his treatment early with a total of 21 Gy given due to his marked clinical response and resolution of symptoms.

**Figure 3:**
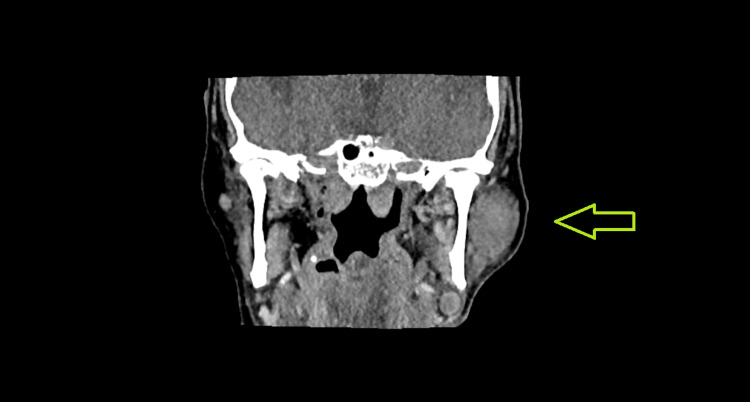
CT showing myeloid sarcoma in the parotid gland.

Two months after his first treatment, he developed a mass on in his parotid gland, which was imaged and later biopsied. The biopsy confirmed a recurrence of his myeloid sarcoma in his parotid gland (Figure 3). Due to the discomfort the mass was causing, another course of 30 Gy over 10 fractions using VMAT was performed to relieve local symptoms. 

## Discussion

This case demonstrates an interesting case of myeloid sarcoma presenting as an ocular mass. As myeloid sarcoma is rare throughout clinical practice and literature, diagnosis remains a challenge. It is often misdiagnosed as abscesses or haematomas as they can present with a clinically similar picture. It is also histologically similar to Hodgkin’s lymphoma and Ewing sarcomas, which can lead to misdiagnosis. Literature suggests that up to 50% of patients with myeloid sarcoma are misdiagnosed [4,5].

Most patients with a diagnosis of myeloid sarcoma but with no concurrent evidence of AML at presentation will go on to develop leukaemia with research estimating the interval to progression of around 10.5 months [6]. The prognosis of myeloid sarcoma is also difficult to predict, with limited data suggesting a median overall survival of six to 14 months if AML is present at diagnosis and 36 months if no evidence of AML is found [7]. The general consensus currently is to treat all cases with intensive chemotherapy, similar to that used to treat AML, regardless of whether there is any detectable leukaemia and even if patients have already undergone resection or radiation. 

Limited literature is suggestive that radiotherapy does not aid overall survival in patients who are also receiving chemotherapy. It has been suggested that radiotherapy can increase the failure-free survival but not the median overall survival [8]. Several studies found nil difference between the survival of patient receiving only chemotherapy and those receiving both chemotherapy and radiotherapy [9,10]. Despite this, as research is limited, it is often still recommended as part of the treatment plan in these patients.

However, even low-dose radiotherapy can achieve excellent local response and control, which can be particularly useful when the myeloid sarcoma is causing local symptoms or in palliation, such as in the case discussed. Myeloid sarcomas are radiosensitive, and complete response rate was found to be 89% where over 30 Gy was used, dropping to 43% when under 20 Gy was used [11]. Therefore, it is recommended to use at least 20 Gy, though some literature suggests that 24 Gy in 12 fractions may be optimal for local control [12].

## Conclusions

This case of a gentleman presenting with an orbital mass, which led to a diagnosis of myeloid sarcoma, highlights the difficulty in diagnosing this rare pathology. Due to its rarity, it is often misdiagnosed, and research surrounding myeloid sarcoma remains limited. Diagnosis of this pathology in a timely manner is critical to aid prompt patient management and prognosis. It is also important to note that it is radiosensitive and low-dose radiation can achieve excellent local control and alleviation of symptoms.
